# Oral Vaccination with Lipid-Formulated BCG Induces a Long-lived, Multifunctional CD4^+^ T Cell Memory Immune Response

**DOI:** 10.1371/journal.pone.0045888

**Published:** 2012-09-25

**Authors:** Lindsay R. Ancelet, Frank E. Aldwell, Fenella J. Rich, Joanna R. Kirman

**Affiliations:** 1 Infectious Diseases Group, Malaghan Institute of Medical Research, Wellington, New Zealand; 2 Immune Solutions Ltd, Centre for Innovation, University of Otago, Dunedin, New Zealand; 3 Department of Microbiology and Immunology, University of Otago, Dunedin, New Zealand; University of Cape Town, South Africa

## Abstract

Oral delivery of BCG in a lipid formulation (Liporale™-BCG) targets delivery of viable bacilli to the mesenteric lymph nodes and confers protection against an aerosol *Mycobacterium tuberculosis* challenge. The magnitude, quality and duration of the effector and memory immune response induced by Liporale™-BCG vaccination is unknown. Therefore, we compared the effector and memory CD4^+^ T cell response in the spleen and lungs of mice vaccinated with Liporale™-BCG to the response induced by subcutaneous BCG vaccination. Liporale™-BCG vaccination induced a long-lived CD4^+^ T cell response, evident by the detection of effector CD4^+^ T cells in the lungs and a significant increase in the number of Ag85B tetramer-specific CD4^+^ T cells in the spleen up to 30 weeks post vaccination. Moreover, following polyclonal stimulation, Liporale™-BCG vaccination, but not s.c. BCG vaccination, induced a significant increase in both the percentage of CD4^+^ T cells in the lungs capable of producing IFNγ and the number of multifunctional CD4^+^ T cells in the lungs at 30 weeks post vaccination. These results demonstrate that orally delivered Liporale™-BCG vaccine induces a long-lived multifunctional immune response, and could therefore represent a practical and effective means of delivering novel BCG-based TB vaccines.

## Introduction

Bacille Calmette-Guérin (BCG) is the only available vaccine for the prevention of tuberculosis [1] and has been given to over 3 billion individuals, making it the most widely administered vaccine to date. BCG is typically administered soon after birth, and while it is effective at preventing TB during childhood, its effectiveness wanes over time [2], [3]. To that end, the efficacy of BCG against adult pulmonary TB is highly variable, ranging from 0–80% [4], [5], [6]. Due to the success of BCG in reducing childhood TB, and its proven safety record, strategies to develop a more effective TB vaccine have focused on improving the efficacy of BCG, either through the development of recombinant BCG strains and attenuated *Mycobacterium tuberculosis* (*Mtb*) vaccine strains or through the development of subunit and virus-vectored vaccines that can be used as a boost for BCG [7]. In that regard, most of the novel TB vaccines currently in the vaccine pipeline are designed to incorporate BCG or attenuated *Mtb* [8].

Optimizing the delivery of this live bacterial vaccine is a further way in which the efficacy of BCG could be improved. Oral delivery of BCG has many advantages over the standard intradermal method of BCG vaccination, including reduced cost, ease of administration, avoidance of needles and the associated risk of disease transfer. More importantly, it has been shown that oral delivery more effectively targets the mucosal immune response than intradermal vaccination [9]. This is critical, given that the primary site of TB infection is the lungs.

BCG is most effective when delivered as a live vaccine [10], [11]. We have previously reported that oral delivery of BCG in a lipid formulation protects the bacilli from degradation in the stomach and provides immunity against an aerosol *Mtb* or *Mycobacterium bovis* challenge in mice and guinea pigs [12], [13], [14], [15]. Moreover, oral BCG vaccination has been shown to boost antigen-specific immune responses in human volunteers [16], [17] and reduce the incidence of virulent *M. bovis* in livestock and wildlife [18], [19], [20], [21].

Immunity to TB is highly dependent upon CD4^+^ T cells and the acquisition of a T helper cell type 1 (Th1) immune response [22]. Control of a primary TB infection is reliant on the production of IFNγ and TNFα in the lungs by CD4^+^ effector T cells [23], [24], [25], [26], [27]. These cytokines activate infected macrophages, enabling them to kill or restrict the growth of the invading mycobacteria [22]. The requirements for a protective memory response to TB are less clear [28]. Lung-resident CD4^+^ T cells appear to be the principal mediators of protection, since following BCG vaccination lung-resident memory T cells have been shown to be sufficient for protection against a mycobacterial challenge [29]. However, the level of IFNγ produced by CD4^+^ T cells is not a reliable predictor of vaccine efficacy [30], [31]. More recently, efforts have focused on measuring the quality of the vaccine-induced immune response by assessing the proportion of multifunctional Th1 CD4^+^ T cells, which are capable of simultaneously producing high levels of IFNγ, TNFα and IL-2 [32]. The increased presence of these cells in the spleens, [33], [34] and perhaps more importantly the lungs, of vaccinated mice has been shown to correlate with protection against TB [35].

Although the vaccine-elicited immune response to parenteral BCG vaccination is well characterized, the CD4^+^ T cell immune response to lipid-formulated oral BCG vaccination is unknown. In this study, we compare the magnitude and quality of the CD4^+^ T cell response in the spleen and lungs of mice induced by orally delivered lipid-formulated BCG to the response induced by subcutaneous (s.c.) BCG vaccination. We report that lipid-formulated oral BCG vaccination (Liporale™-BCG) induced a long-lived CD4^+^ T cell response, demonstrated by the persistence of activated, antigen-specific CD4^+^ T cells in the spleens and a greater number of multifunctional CD4^+^ T cells in the lungs than s.c. vaccinated mice at 30 weeks post immunization. These findings suggest that Liporale™-BCG vaccination could present an effective means of delivering novel BCG-based vaccines for the prevention of TB.

## Materials and Methods

### Ethics Statement

This project was undertaken within the provisions of the Animal Welfare Act (1999) of New Zealand and was approved by the Victoria University of Wellington Animal Ethics Committee.

### Mice

Inbred C57BL/6 mice were purchased from The Jackson Laboratory and bred and housed under SPF conditions at the Malaghan Institute of Medical Research Biomedical Research Unit in Wellington, New Zealand. Groups of 5 age- and sex-matched mice were used at each time point for each experimental group.

### BCG Preparation and Vaccination

Mice were vaccinated per oral or subcutaneous route, as indicated, with *M. bovis* BCG Danish strain 1331. BCG was grown to mid log-phase in 175 ml flasks (Falcon, NJ, USA) containing Middlebrook 7H9 medium (Difco, Detroit, MI, USA) supplemented with albumin-dextrose-catalase (BBL, Becton Dickinson, MD, USA) and 0.01% Tween 80. BCG in vaccine preparations was enumerated by plating onto modified Middlebrook 7H11 agar (Difco, Detroit, MI, USA) containing oleic acid-albumin-dextrose-catalase ((BBL, Becton Dickinson, MD, USA) and glycerol and counting retrospectively after incubation for 2–3 weeks. For formulating the oral vaccine, broth-grown BCG bacilli were pelleted by centrifugation and encapsulated into Liporale™ as previously described (13). For subcutaneous vaccination, 50 uL of 7H9 medium, containing approximately 1×10^6^ CFU BCG, was injected into the right flank. For oral BCG administration, mice were temporarily separated into individual cages and offered 0.3mL chocolate-flavored Liporale™ containing 1–2×10^7^ CFU BCG. After 12 hours, the oral vaccine had been entirely consumed and mice were placed back in their original cages.

### Tissue Preparation

At times indicated, mice were culled by cervical dislocation. Single lymphocyte suspensions were prepared from spleens of mice by passing them through a 70 µm cell strainer and subjecting them to red blood cell lysis (Red Blood Cell Lysing Buffer, Sigma, St. Lois, MO). Lung lymphocytes were isolated by enzymatic digestion of lung tissue (2.4 mg/mL Collagenase Type I (Invitrogen, Carlsbad, CA), 0.12 mg/mL DNase 1 (Roche, Mannheim, Germany) in Iscove’s Modified Dulbecco’s Medium (IMDM) without additives), and CD45^+^ cells were isolated using magnetic bead enrichment with CD45 MicroBeads (MACS, Miltenyi Biotec, Germany). Total cell counts per organ were determined using a haemocytometer following red blood cell lysis of spleens or following CD45 MicroBead isolation from lungs.

### In Vitro Restimulation

Single cell lymphocyte suspensions from spleens and lungs were plated at a density of 4×10^6^/mL in a 24 well plate and incubated for 6 hours at 37°C in IMDM (supplemented with 5% FCS, 1% penicillin/streptomycin, 1% L-glutamine (GlutaMAX, Gibco, Invitrogen, Auckland, New Zealand), 0.1% 2-mercaptoethanol (Gibco, Invitrogen)) containing 2 µg/mL anti-CD3 (clone 2C11) and 2 µg/mL anti-CD28 (clone 37.51) (both prepared in house). 3 µg/mL Brefeldin A (eBioscience, San Diego, CA) and 2 µM monensin (Sigma) were added for the last 4 hours of incubation.

### Identification of Tetramer-specific Cells

Single cell suspensions from spleens were stained with I-A(b) *Mtb* antigen 85B precursor 280–294 (FQDAYNAAGGHNAVF) tetramer-APC or with I-A(b) human class II-associated invariant-chain peptide (PVSKMR MARPLLMQA) tetramer-APC as a negative control (NIH MHC Tetramer Core Facility at Emory University, Atlanta, GA), and enriched by positive magnetic bead isolation using the AutoMACS cell sorter (Miltenyi Biotec) following staining with anti-APC MicroBeads (MACS, Miltenyi Biotec) and were identified by flow cytometry. For phenotype analysis we used a minimum number of 50 events in the tetramer gate, with a mean of 180 events.

### Flow Cytometry

Lymphocytes were labeled with anti-CD4-Pac Blue, (BD Biosciences, San Diego, CA), anti-CD44-PE-Cy7, anti-CD62L-APC-AlexaFluor 750 (both from eBioscience) for cell surface staining, and anti-IFNγ-PE-Cy7, anti-IL2-APC (both from eBioscience), and anti-TNFα-PE (BD Biosciences) for intracellular staining. Dead cells were excluded following staining with the viability dye LIVE/DEAD® Fixable Blue Dead Cell Stain (Invitrogen). All samples were collected on a BD LSRII SORP (Becton Dickinson, San Jose, CA) and FlowJo software version 9.4 was used for data analysis.

### Statistical Analysis

Statistical significance was determined by one-way ANOVA followed by the Tukey post-test, two way ANOVA followed by the Bonferroni post test, or by the Mann Whitney test, as indicated in figure legends, using Prism software.

**Figure 1:**
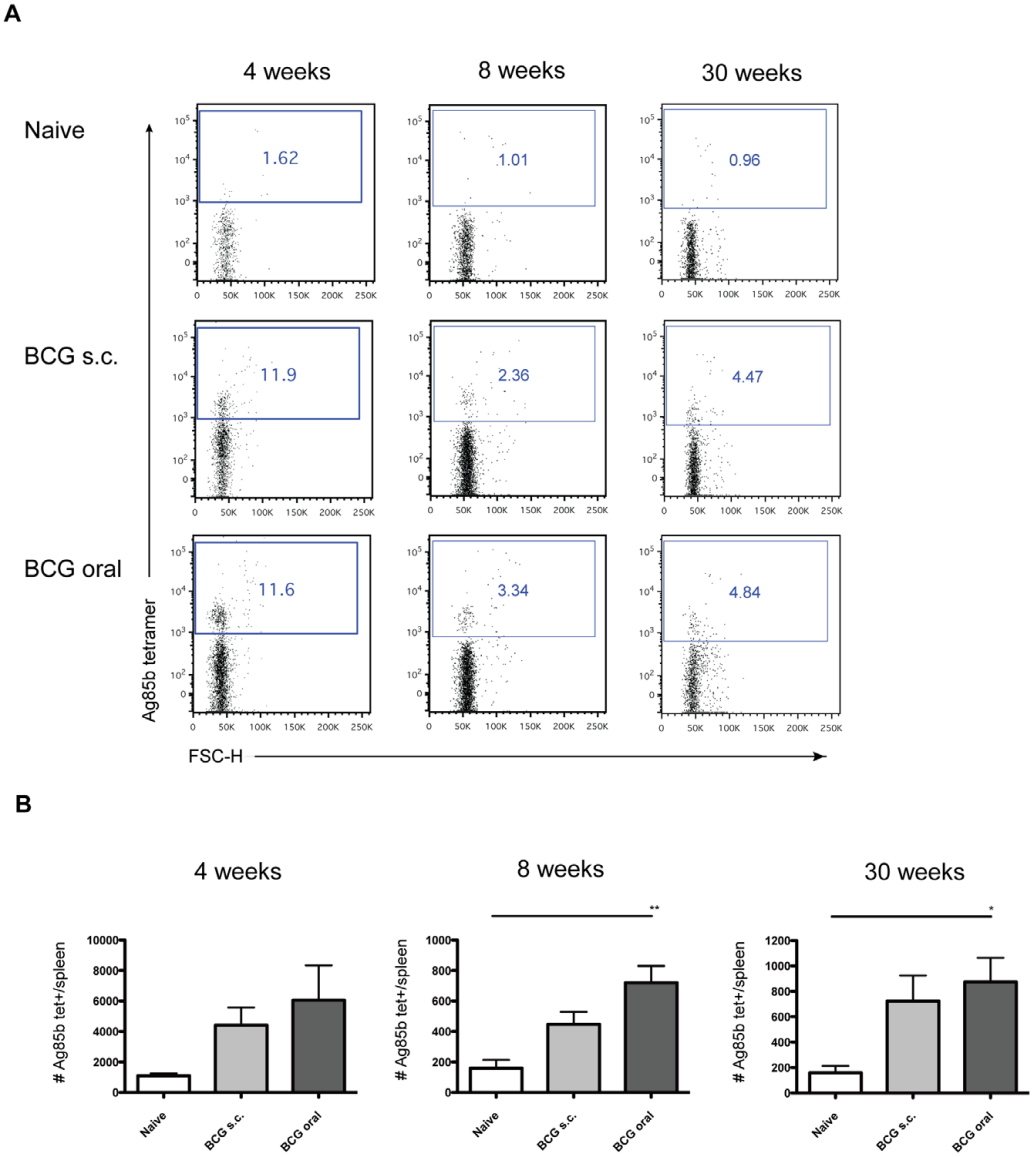
Oral vaccination with Liporale™-BCG increases the number of Ag85B-specific CD4^+^ T cells in the spleen. Lymphocytes from the spleens of naïve, Liporale™-BCG vaccinated (BCG oral) or subcutaneously vaccinated (BCG s.c.) mice were stained with an Ag85B/MHCII tetramer and enriched for tetramer positive cells by magnetic bead isolation. (A) Representative flow cytometry plots show Ag85B-specific CD4^+^ T cells in the spleens of naïve or BCG vaccinated mice at 4, 8 and 30 weeks post immunization. (B) Bar graphs show the number of Ag85B-specific CD4^+^ T cells in the spleens of naïve or BCG vaccinated mice at 4, 8 and 30 weeks post vaccination. Results are displayed as mean +SEM of n = 5 for each group, significance expressed relative to naïve: *p<0.05, **p<0.01, ***p<0.001 (one way ANOVA with Tukey post test). The 8 and 30 weeks results are representative of 2 independent experiments.

**Figure 2:**
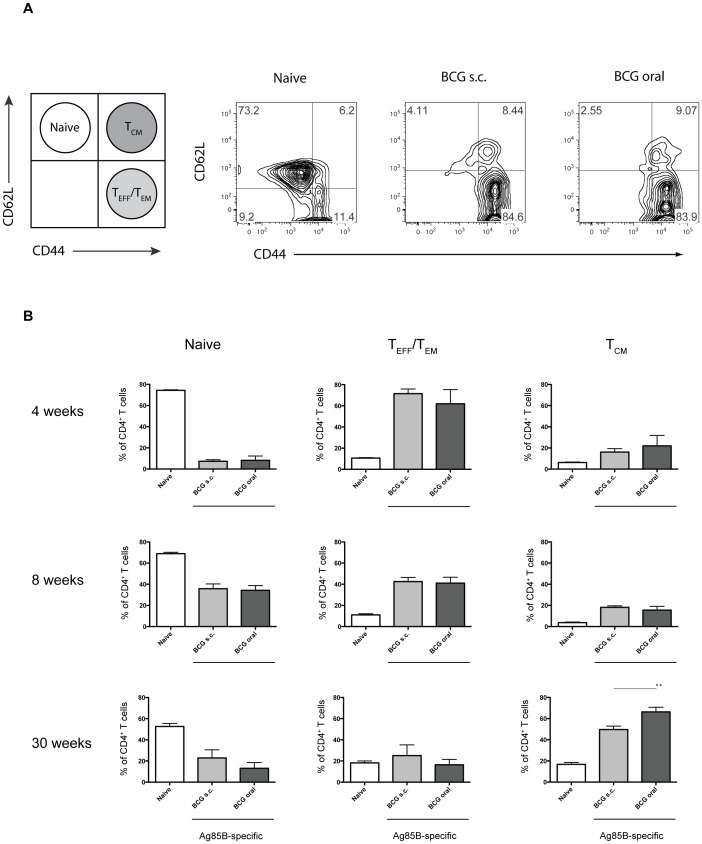
Oral vaccination with Liporale™ BCG induces effector and central memory Ag85B-specific CD4^+^ T cells in the spleen. Lymphocytes from the spleens of naïve, Liporale™-BCG vaccinated (BCG oral) or subcutaneously vaccinated (BCG s.c.) mice were stained with an Ag85B/MHCII tetramer and enriched for tetramer positive cells by magnetic bead isolation. (A) Representative flow cytometry density plots showing CD62L and CD44 expression on total CD4^+^ T cells from spleens of naïve mice, or Ag85b-specific CD4^+^ T cells from the spleens of BCG vaccinated mice. (B) Bar graphs showing the proportion of naïve (CD62L^hi^, CD44^lo^), T_EFF_/T_EM_ (CD62L^lo^, CD44^hi^) or T_CM_ (CD62L^hi^, CD44^hi^) CD4^+^ T lymphocytes of total CD4^+^ T cells from naïve mice or Ag85B-specific CD4^+^ T cells from the spleens of BCG vaccinated mice at 4, 8 and 30 weeks post vaccination. Results are displayed as mean + SEM of n = 5 for each group: *p<0.05, **p<0.01, ***p<0.001 (Mann-Whitney test). Eight and 30 weeks results are representative of 2 independent experiments.

## Results

### Liporale™-BCG Significantly Increases the Number of Ag85B-specific CD4^+^ T Cells in the Spleen

To compare the immune response elicited by Liporale™-BCG to the response induced by s.c. BCG vaccination, we isolated lymphocytes from the spleens of vaccinated C57Bl/6 mice at 4, 8 and 30 weeks post immunization. Naïve mice served as unvaccinated controls. Splenic CD4^+^ T cells specific for the immunogenic BCG and *Mtb* antigen Ag85B were identified using an MHCII-Ag85B tetramer (Fig. 1a). Importantly, only mice that received Liporale™-BCG had a significant increase in the number of Ag85B-specific cells in the spleen compared to naïve controls at 8 and 30 weeks following immunization (Fig. 1b).

**Figure 3:**
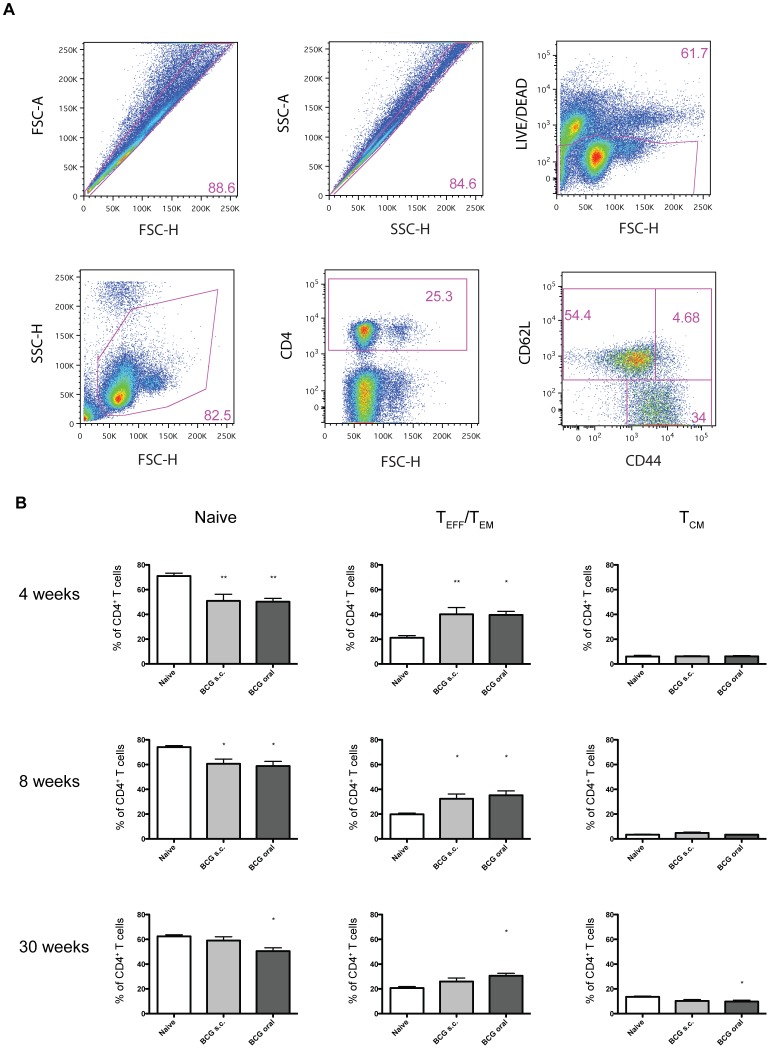
Oral vaccination with Liporale™-BCG induces long-lived CD4^+^ effector T cells in the lung. Lymphocytes from the lungs of naïve, Liporale™-BCG vaccinated (BCG oral) or subcutaneous vaccinated (BCG s.c.) mice were analyzed by flow cytometry. (A) Representative flow cytometry plots show the gating strategy used to identify CD4^+^ T cells. (B) Bar graphs show the proportion of naïve (CD62L^hi^, CD44^lo^), T_EFF_/T_EM_ (CD62L^lo^, CD44^hi^) or T_CM_ (CD62L^hi^, CD44^hi^) CD4^+^ T lymphocytes of total CD4^+^ T cells from the lungs of naïve or BCG vaccinated mice at 4, 8 and 30 weeks post vaccination. Results are displayed as mean + SEM of n = 5 for each group, significance expressed relative to naïve: *p<0.05, **p<0.01, ***p<0.001 (one way ANOVA with Tukey post test). Eight and 30 weeks results are representative of 2 independent experiments.

### Liporale™-BCG Induces an Ag85B-specific CD4^+^ Effector T Cell Phenotype in the Spleen

To distinguish between naïve, effector (T_EFF_)/effector-memory (T_EM_) and central memory (T_CM_) CD4^+^ T cell populations, total CD4^+^ lymphocytes from naïve mice or Ag85B-specific CD4^+^ T cells from vaccinated mice, were stained for the expression of CD62L, a lymphoid homing receptor that is downregulated shortly following T cell activation, and the activation marker CD44. CD4^+^ T cell subsets were categorized as follows: naïve cells as CD62L^hi^, CD44^lo^; T_CM_ as CD62L^hi^, CD44^hi^ and T_EM_/T_EFF_ as CD62L^lo^, CD44^hi^ [36], [37].

At 4 weeks post immunization, the Ag85B-specific CD4^+^ T cells from the spleens of mice vaccinated with Liporale™-BCG or s.c. BCG displayed a predominantly T_EM_/T_EFF_ phenotype (Fig. 2 a and b). The antigen-specific CD4^+^ effector immune response was maintained up to 8 weeks post immunization, illustrating that Liporale™-BCG can induce an antigen-specific CD4^+^ effector T cell response in the spleen that is equivalent to s.c. BCG vaccination.

**Figure 4:**
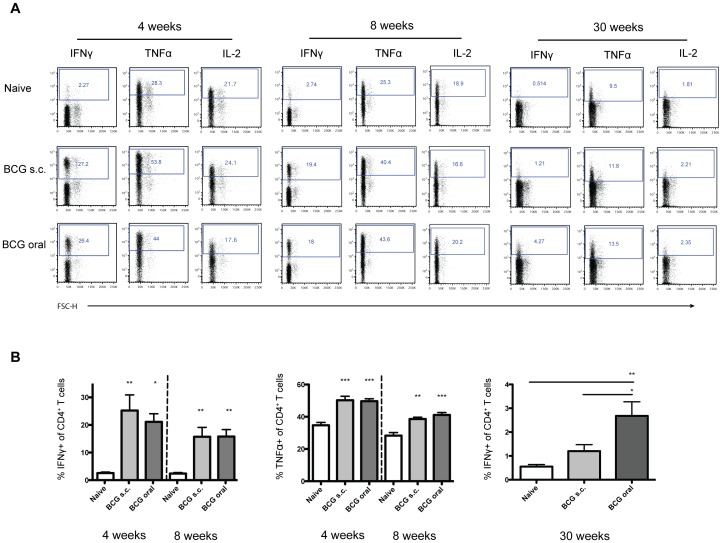
Oral vaccination with Liporale™-BCG induces CD4^+^ T cell cytokine production in the lung. Lymphocytes from the lungs of naïve, Liporale™-BCG vaccinated (BCG oral) or subcutaneous vaccinated (BCG s.c.) mice were stimulated for 6 hours *in vitro* in the presence of Brefeldin A and monensin then analyzed by flow cytometry. (A) Representative plots show CD4^+^ T cells from the lungs of naïve or BCG vaccinated mice expressing IFNγ, TNFα or IL-2. (B) Bar graphs show the percentage of CD4^+^ T cells from the lungs of naïve or BCG vaccinated mice expressing cytokines at 4, 8 or 30 weeks post immunization. Results are displayed as mean + SEM of n = 5 for each group, significance expressed relative to naïve: *p<0.05, **p<0.01, ***p<0.001 (one way ANOVA with Tukey post test). Eight and 30 weeks results are representative of 2 independent experiments.

By 30 weeks post immunization, >50% of the Ag85B-specific CD4^+^ T cells in the spleens of vaccinated mice were T_CM_, consistent with reports demonstrating that central memory cells are capable of long term survival [38]. Interestingly, there was a significant increase in the proportion of T_CM_ cells in mice that received BCG orally compared to mice that received a s.c. BCG immunization (p<0.05), suggesting that Liporale™-BCG vaccination can induce a long-lived antigen specific memory immune response.

**Figure 5:**
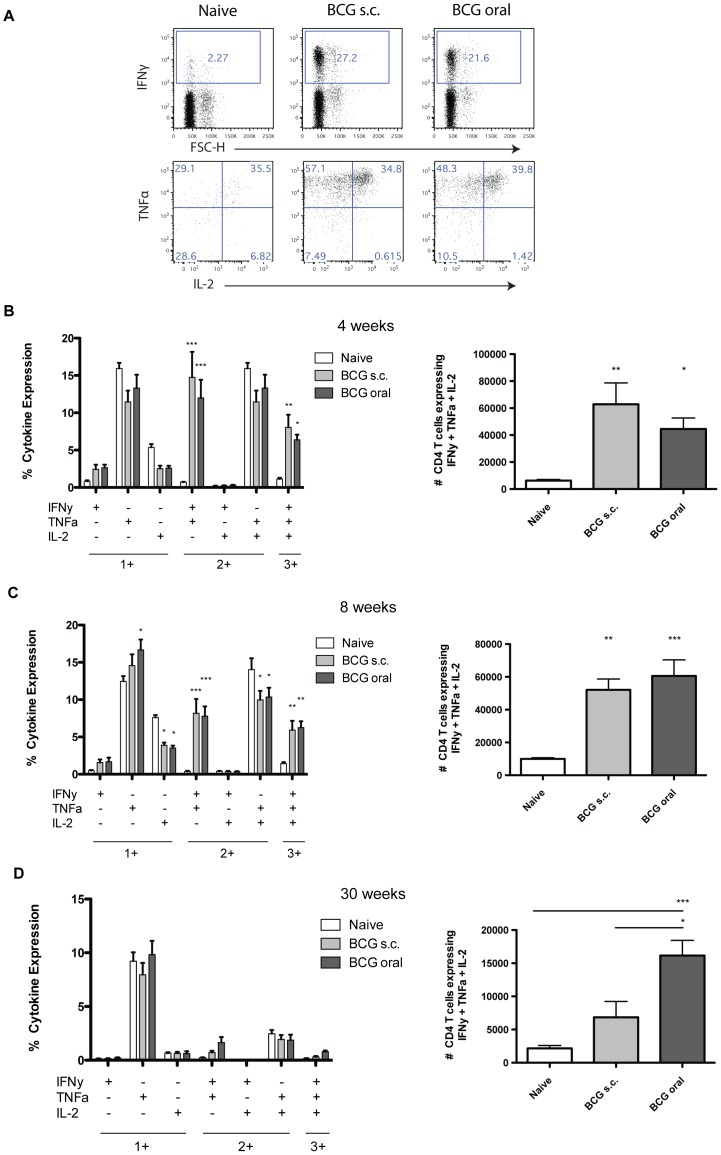
Increased multifunctional CD4^+^ T cells in the lungs of Liporale™-BCG vaccinated mice. Lymphocytes from the lungs of naïve, Liporale™-BCG vaccinated (BCG oral) or subcutaneous vaccinated (BCG s.c.) mice were stimulated for 6 hours in the presence of Brefeldin A and monensin then analyzed by flow cytometry. (A) Representative dot plots of CD4^+^ T cells from the lungs of naïve or BCG vaccinated mice expressing IFNγ, TNFα or IL-2 cytokines. Multifunctional cells were identified by first gating on IFNγ+ cells, and then on cells positive for both TNFα and IL-2. (B) Bar graph showing the percentage of CD4^+^ T cells from naïve or BCG vaccinated mice producing 1, 2 or 3 cytokines simultaneously, and bar graph of the frequency of multifunctional CD4^+^ T cells in the lungs of naïve or vaccinated mice at 4 weeks post immunization. (C,D) Bar graphs as in (B), but showing results for 8 and 30 weeks post vaccination, respectively. Results are displayed as mean + SEM of n = 5 for each group, significance expressed relative to naïve: *p<0.05, **p<0.01, ***p<0.001 (two way ANOVA with Bonferroni post test (Figure 5a, b and c, left graphs), one way ANOVA with Tukey post test (Figure 5a, b and c, right graphs)). Eight and 30 weeks results are representative of 2 independent experiments.

### Liporale™-BCG Induces a Long-lived CD4^+^ Effector T Cell Phenotype in the Lungs

To investigate the immune response to lipid-formulated oral BCG vaccination in the lung, we isolated lymphocytes from the lungs of mice 4, 8 and 30 weeks post oral or subcutaneous BCG vaccination. We determined the phenotype of the total CD4^+^ T cell population using antibodies against CD62L and CD44 to distinguish between naïve, T_EM_/T_EFF_ and T_CM_ subsets (Fig. 3a). Similar to the antigen-specific response in the spleen, at 4 and 8 weeks post immunization, Liporale™-BCG induced a T_EM_/T_EFF_ CD4^+^ T cell phenotype in the lungs that was equivalent to the immune response observed when BCG was administered subcutaneously (Fig. 3b). Importantly, by 30 weeks following immunization, only mice that received Liporale™-BCG maintained a CD4^+^ T_EM_/T_EFF_ phenotype relative to naïve controls. The maintenance of a CD4^+^ T_EM_/T_EFF_ cell population in the lungs of orally vaccinated mice 7 months after immunization suggests that oral BCG vaccination induces a prolonged effector response in the lungs compared to subcutaneous vaccination.

### Cytokine Production by CD4^+^ T Cells from the Lungs of Liporale™-BCG Vaccinated Mice

To assess the quality of the immune response in the lung following Liporale™-BCG vaccination, lymphocytes were isolated from the lungs of naïve or BCG-vaccinated mice and restimulated for 6 hours *in vitro* in the presence of Brefeldin A and monensin. Cytokine producing CD4^+^ T cells were identified by intracellular staining and flow cytometry. At 4 and 8 weeks after vaccination, there was a significant increase in the proportion of CD4^+^ T cells producing IFNγ or TNFα, but not IL-2, in the lungs of mice that received either Liporale™-BCG or s.c. BCG vaccines compared to naïve controls (Fig. 4 a and b). Of note, by 30 weeks post immunization, there was a significant increase in the proportion of IFNγ-producing CD4^+^ T cells from the lungs of mice that received Liporale™-BCG vaccination relative to cells from s.c. BCG vaccinated mice and naïve controls (Fig. 4 a and b).

The frequency of multifunctional CD4^+^ T cells in the lungs of vaccinated and naïve mice was determined using Boolean gating. At 4 and 8 weeks after vaccination with either Liporale™-BCG or s.c. BCG there was a significant increase in the percentage and number of multifunctional CD4^+^ T cells in the lungs of mice compared to cells from naïve controls (Fig. 5 a, b and c). The proportion of IFNγ^+^TNFα^+^IL-2^−^ double positive CD4^+^ T cells also was also significantly increased in the lungs of Liporale™-BCG or s.c. BCG vaccinated mice compared to naïve controls (Fig. 5 b and c). Interestingly, at 30 weeks after immunization, there was a significant increase in the frequency of multifunctional CD4^+^ T cells in the lungs of mice that received Liporale™-BCG compared to s.c. vaccinated or naïve controls (Fig. 5d).

## Discussion

Oral delivery of live BCG using a lipid formulation has been shown to be effective at protecting animals against a virulent mycobacterial challenge [12], [14], [15], [39]; however, the immune response elicited by this vaccine had not been fully investigated. Therefore we compared the CD4^+^ T cell response in mice vaccinated with Liporale™-BCG to mice vaccinated with BCG through the conventional s.c. route. We observed a significantly increased number of Ag85B tetramer-specific CD4^+^ T cells in the spleens of mice vaccinated with Liporale™-BCG and found that oral vaccination induced T_EFF_/T_EM_ and T_CM_ CD4^+^ T cell populations in the spleen that were similar to s.c. BCG vaccinated mice. Moreover, following polyclonal stimulation, we found that mice vaccinated with Liporale™-BCG had significantly more IFNγ-producing, and multifunctional CD4^+^ T cells in the lungs, the primary site of TB infection, than s.c. BCG vaccinated or control mice >6 months after vaccination.

The earlier control of bacterial growth observed following pulmonary mycobacterial challenge of memory immune mice has been shown to coincide with the early arrival of antigen-specific Th1 CD4^+^ T cells in the lungs [40]. Supporting this, it has been demonstrated that adoptively transferred activated, transgenic Th1 ESAT-6-specific cells traffic to the lungs and protect from an *Mtb* challenge in a frequency dependent manner [41]. Moreover, using FTY720 to block egress of lymphocytes from the lymph nodes, it has been shown that T cells in the lungs of BCG vaccinated mice are sufficient to protect against a mycobacterial challenge [29]. Together, these studies suggest that the presence of Th1 CD4^+^ T cells in the lungs is critical for the immune protection afforded by vaccination. We found that both Liporale™-BCG vaccination and s.c. BCG vaccination led to a statistically significant increase in the frequency of IFNγ-producing CD4^+^ T cells in the lungs at 4 and 8 weeks after immunization, which coincided with the presence of a predominantly effector phenotype of the CD4^+^ T cells in the lungs. By 30 weeks post vaccination, a significant population of T_EFF_/T_EM_ CD4^+^ T cells capable of producing IFNγ was identified only in the lungs of mice that were vaccinated with Liporale™-BCG, suggesting that the oral route of vaccination produces a more sustained immune response in the lungs than traditional s.c. vaccination.

Although IFNγ is necessary to control an *Mtb* infection, the IFNγ response induced by TB vaccination is an unreliable correlate of vaccine-elicited protection [30], [31]. For this reason we also assessed the frequency of multifunctional cells in the spleens and lungs of vaccinated mice, since these cells have been shown to correlate with vaccine-elicited protection from *Mtb* in mice [32], [33], [35]. We found an increase in the proportion and frequency of triple cytokine producing cells in the lung at 4 and 8 weeks in mice vaccinated with BCG orally or s.c., but interestingly, only Liporale™-BCG maintained a significant population of multifunctional CD4^+^ T cells in the lungs of mice by 30 weeks post vaccination. Therefore, the oral, mucosal vaccination route maintains the multifunctional CD4^+^ T cell population in the lung for longer than the traditional s.c. route of immunization.

By contrast, Kaveh *et al*. recently reported that intradermal BCG vaccination induces a long-lived population of multifunctional CD4^+^ T cells in the lungs [42]. In this study an increase in the proportion of multifunctional cells in vaccinated mice compared to naïve controls was detected at 6 weeks post immunization, however, it is unclear whether the percentage of multifunctional cells in BCG vaccinated mice were above that found in naïve mice at 6, 12 and 18 months after vaccination because unvaccinated controls were not included at later time points. The phenotype of the multifunctional cells in the spleens of intradermally BCG vaccinated mice was reported as T_EM_–like (CD44^hi^, CD62L^lo^); however, it is important to note that this phenotype was assessed after an 18 hour *in vitro* restimulation, and it is likely that the phenotype of these cells was altered by the restimulation [43].

In our study we found that Ag85B tetramer-specific CD4^+^ T cells in the spleen maintained an effector phenotype up to 8 weeks following either s.c. or Liporale™-BCG vaccination. By contrast, at 30 weeks post vaccination, over 50% of the antigen-specific CD4^+^ T cells in the spleens of vaccinated mice had a T_CM_ phenotype, with a significantly higher proportion of T_CM_ in the orally vaccinated mice. Similar to the early response in the spleen, we observed a pool of CD4^+^ T_EFF_/T_EM_ cells at 4 and 8 weeks in the lungs of mice that received BCG either orally or subcutaneously. This population of CD4^+^ T_EFF_/T_EM_ cells was maintained up to 30 weeks post Liporale™-BCG vaccination. It is possible that the low level of persisting antigen in the lungs following oral BCG vaccination contributed to the maintenance of T_EFF_/T_EM_ cells in the lung up to 6 months after vaccination; however it should be noted that an earlier study demonstrated that viable bacteria could not be recovered from the lungs by 8 weeks following oral BCG vaccination [44].

A further possible mechanism for the extended presence of T_EFF_/T_EM_ lymphocytes following oral BCG vaccination is tissue-specific homing, in which T cells preferentially migrate to the tissues in which they were primed [45]. There is evidence that T cells primed in mucosal lymphoid sites, such as the mesenteric lymph nodes and Peyer’s patches, express homing markers and chemokines specific for the major mucosal sites in the body, the lung and the intestine [46], [47], [48]. Following Liporale™-BCG vaccination, live mycobacteria are predominantly found in mucosal lymphatic tissues, such as the mesenteric lymph nodes, cervical lymph nodes and the Peyer’s patches, and is therefore thought to be where T cell priming occurs [44], [49]. Interestingly, lymphocytes isolated from the spleen of mice vaccinated with Liporale™-BCG did not express the mucosal homing molecules CD103 or α4β7, but differentially expressed β1 integrin, which has been shown to be involved in T cell homing to the lung epithelium [49], [50], [51]. Whether the antigen-specific CD4^+^ T cells recovered from the spleens of Liporale™-BCG vaccinated mice express β1 integrin remains to be determined.

We have shown previously that Liporale™-BCG vaccination effectively protects against *Mtb* infection and in this present study we have provided evidence that orally delivered, Liporale™-BCG vaccination induces a strong antigen-specific CD4^+^ T_EFF_/T_EM_ and T_CM_ response, which appears superior to s.c. BCG vaccination. Due to the ability of BCG to protect against childhood TB, most TB vaccines currently in clinical trial incorporate either recombinant BCG, attenuated *Mtb* strains or boosting regimes to maintain this protection [8]. Given the long-lived immune response we see in the lungs of mice following Liporale™-BCG vaccination, we speculate that the delivery of novel live TB vaccines via the oral route may more effectively target the mucosal immune response than traditional immunization routes, enhancing protection against aerosol *Mtb* infection.
